# Hospital and Institutional News

**Published:** 1916-04-22

**Authors:** 


### HOSPITAL AND INSTITUTIONAL NEWS.

### THE LATE B. BURFORD RAWLINGS.

We regret to have to report the death, on April 16, of Mr. B. Burford Rawlings, a one-time famous and most conscientious hospital secretary. He was the son of the late Mr. B. W. Rawlings, of John Street, Bedford Row, and Romford House, Romford. In 1866 he was appointed secretary to the National Hospital for the Paralysed and Epileptic, founded in 1859 for the treatment of nervous diseases, for which there was at this time no provision whatever. Mr. Rawlings made the hospital his sole object, and subordinated his personal interests to its welfare. When the new buildings (the Albany Memorial) were completed in 1885, he was given the additional title of General Director; and, mindful of his single-hearted services, the Board of Management put it on record that the building fund "had been raised and administered by gratuitous labour." Mr. Rawlings was universally respected, and his influence was considerable, for his pen was one of the ablest ever wielded by a hospital secretary, his style being dignified and often displaying distinct literary charm. He had a wide knowledge of hospital matters, and one result of his able and continuous work was to add largely to the volume of subscriptions to all hospitals. Mr. Rawlings for some years before his enforced retirement had been underpaid and overworked, so that he had no time, and, indeed, no thought for himself. A recognition of these facts contributed to the decision of the governors to grant him one year's full salary in recognition of his special services, in addition to a pension of £450 per annum. This pension will be seen to have been none too liberal when it is remembered that his salary was only £600, and that upon Mr. Rawlings devolved the duty of creating and working building funds, special begging appeals, and all the business of the hospital, in addition to its superintendence and administration. His faithfulness as a guardian of the hospital's estate was shown by the ability with which he gradually acquired adjoining properties which, when consolidated, provided an extensive site for the erection thereon of the hospital as it exists to-day. He was a tough and courageous fighter in matters of principle. His uncompromising insistence on the supremacy of lay control in hospital administration presently brought him into conflict with the medical staff, and a long controversy (see The Hospital, December 21, 1901, pages 193 and 194, 205-207, and previous volumes) between the Board of Management, with Mr. Rawlings as protagonist, and the medical staff, resulted in the resignation in 1901 both of the Board and of its secretary. Mr. Rawlings thus brought to a close his thirty-five years' service with the National Hospital. He was requested to remain in office during many months whilst the governors were settling their differences, a fact which testifies to his value and mastery of the affairs of his hospital and its maintenance. In his days of activity he contributed articles to leading reviews. He published some volumes of verse which were dealt with in The Hospital of February 26, 1916, p. 469. He often contributed to The Hospital, his latest effort being "The Evolution of the Modern Hospital" (see vols. lvii. and lviii., February to June, 1915). Mr. Rawlings has left a widow, one son, and a daughter to mourn his loss. His last thoughts were for his old hospital, for so recently as 30th ult. he wrote: "Illness makes writing difficult, but I cannot neglect to offer an expression of true sympathy for the hospital and all its workers, in these anxious days."

### THE INSPECTOR OF MILITARY ORTHOPÆDICS.

The War Office having decided to institute an Inspector of Military Orthopædics, there could be little doubt in the minds of anyone upon whom the first choice would fall—namely, upon Mr. Robert Jones, of Liverpool. Colonel Jones, to give him his less familiar military title, has accepted the appointment, and his duties will be to exercise general supervision on behalf of the War Office in respect of the treatment of orthopædic cases in the various military hospitals and sections of hospitals set apart for them; and to arrange for the transfer of suitable cases to selected centres for orthopædic treatment. The Hammersmith Military Hospital, it is understood, will be the first of these orthopædic hospitals to be organised under Colonel Jones' supervision. The buildings consist of the Hammersmith Infirmary and Workhouse, and will accommodate 800 patients. There will be departments for massage, mechano-therapy, electrical treatment, radiant heat and whirlpool baths, as well as for the operative surgical treatment which will constitute so important a part of the hospital's work. The workshops of the building are large, and it is proposed to fit them up in such a way that all splints, surgical boots, plaster-of-Paris casts, and appliances generally will be made upon the premises. When these buildings were erected much criticism of their lavish appointments was levelled at the Guardians; but as things have turned out no one will now grudge the money which was then spent, for it means more comfort for a class of soldier who has peculiar claims upon the sympathy of his fellow-countrymen.

### THE MILITARY HEART HOSPITAL AT HAMPSTEAD.

That peculiar source of military wastage and disability known as "soldier's heart" was not unknown in the Army in peace time; but naturally enough it is far more prominent now, as the experience of past campaigns plainly enough showed would probably happen. Whether this complaint has wholly or mainly a single definite cause, or whether it is merely a train of symptoms which may arise from many different causes, is still not finally decided; but the arrangements which the Army Medical Service has made for the treatment of soldiers suffering from it in the military hospital at Hampstead, and the facilities for research there by competent scientific workers, should result in considerable acquisitions of knowledge concerning the ætiology as well as the treatment of this malady. Very soon after the outbreak of war—in the early winter of 1914, that is to say—the number of cases was already such that special instructions about them were issued to the large hospitals in France; and special wards were set aside in many hospitals for the isolation of these patients so that the prohibition of tobacco could be enforced. Whatever the result of Sir James Mackenzie's researches may be, one may confidently expect that the toxic effects of excessive tobacco-smoking will be found to be one of the factors of "soldier's heart."

### A CASE OF LEPROSY IN LONDON.

Some astonishment seems to have been created by the evidence given at an inquest on April 12 on a Cingalese medical student who died in London of leprosy. Leprosy, as readers of The Hospital are probably aware, is by no means unknown in England; though all the cases, so far as is known, are imported, and have not spread infection amongst our population. Only two or three years ago a special hostel for such cases was started, and this attempt at voluntary segregation is a considerable safeguard against contagion. In the case which formed the occasion of the recent inquest, the patient had been in England for six years, but had been a leper for nineteen years. It seems extraordinary that a man who had already been a leper thirteen years should come to England and commence a medical training. As long as aliens suffering from contagious and deadly diseases are allowed to land unmolested and uninspected on our shores (in the sacred name of liberty, of course), it is difficult to see any way by which a repetition of the event is to be avoided.

### STATE-AIDED TREATMENT OF VENEREAL DISEASES.

The National Council for Combating Venereal Diseases is evidently, and rightly, determined to strike while the iron is hot. A deputation has waited upon the President of the Local Government Board and received a most sympathetic hearing from him. Mr. Long informed the deputation that he had communicated with the Treasury, and they are prepared to provide the necessary grant for carrying out the recommendations of the Royal Commission with regard to the provision of facilities for diagnosis and treatment. These grants would cover 75 per cent. of the cost incurred by local authorities. The Board does not favour the policy of starting separate hospitals for these diseases, but proposes to utilise the general hospitals. In this the department is wise, and it only remains for local authorities and voluntary hospitals to rise to the occasion. The other major recommendations of the Royal Commission cannot be carried into effect by departmental action alone, but require legislation; such suggestions, for example, as the confidential registration of causes of death, legal protection for medical men making communications concerning those about to marry, and the constitution of venereal disease as a statutory incapacity for a contract of matrimony.

### M.P.S' SALARIES FOR HOSPITALS.

The fashion set by a handful of members of Parliament at the time when the House of Commons first voted a salary for every one of its members—of handing over the sum to a hospital—has not died out with the impulse which produced it. Only last week the general superintendent of King Edward VII.'s Hospital, Cardiff, Mr. L. D. Rea, received £43 3s. 6d. from Mr. J. Herbert Cory, M.P. The donor explained that this sum was half the amount he had received as the salary of a member of Parliament, and that he was sending the remaining half to the (local) Seamen's Hospital. With Mr. Cory's political motive we are not concerned, but what is so gratifying is that, when men for any reason are troubled as to the best way of disposing of any sums of money, the voluntary hospital at once suggests itself to their minds as an unimpeachable means of devoting it to the public good. To have achieved this, to have made hospitals, as it were, the residuary legatee of all sums likely to be spent on charity is the best proof of the strength of the voluntary system in the affections of our countrymen.

### TAXATION OF DOCTORS' CARS.

At a meeting of the Executive Committee of the National Medical Union, held at 346 Strand on April 13, 1916, the following resolution was passed: "That, owing to the great amount of public work, both military and civil, now being done by medical men, and the use of the motor as a means of accomplishing that work being indispensable, it is urged by the National Medical Union that no further burden of taxation be imposed upon the medical profession, either in connection with the proposed increased taxation of motor-cars and motor-cycles or with reference to the taxation or limitation of the supply of petrol." It was also resolved that a copy of this resolution be sent to the Chancellor of the Exchequer.

### A GIFT OF LAND TO THE ROYAL FREE HOSPITAL.

At the last Court of Governors of the Royal Free Hospital, Gray's Inn Road, Sir Richard Stapley announced that an anonymous benefactor has presented nearly an acre of land adjoining the hospital. This land will be available, it is stated, for extensions particularly needed in connection with maternity work and infant welfare. The income derived from the present tenancies is to be specially allocated towards the cost of preparing a scheme for the development of the site. This self-denying ordinance is a most excellent idea, for it ensures that there shall be no slackening of effort towards obtaining the funds necessary for the normal working of the hospital, which might occur if the idea got about that the Royal Free Hospital had been endowed with a number of rents by the very practical generosity of this munificent donor.

### "NAMED BEDS" PRODUCTIVENESS IN SCOTLAND.

Subscriptions are rolling in merrily for the naming of beds in Scottish Red Cross hospitals. Up to date there are no fewer than 1,012 named beds, distributed as follows:—477 in the Scottish National Red Cross Hospital; 309 in the Scottish Hospital at Rouen; 202 in the Springburn and Woodside Hospitals; 24 in l'Hôpital de l'Ecosse in Paris.

### THE SCOTTISH HOSPITAL FOR LIMBLESS WARRIORS: £52,000 IN A MONTH.

Up to last week-end over £52,000 had been received by the Lord Provost of Glasgow in subscriptions towards the Princess Louise Scottish Hospital for Limbless Sailors and Soldiers at Erskine. This is a grand result. Among the latest donations are:—£1,000 from the Bellahouston Bequest Fund Trustees, Glasgow; £500 from Messrs. David Colville and Sons, Limited, Motherwell; £250 each from Messrs. William Graham and Co., Glasgow, "Anonymous," and Nobel's Explosives Co., Ltd., Glasgow; £200 each from Miss Lithgow, Drums, Langbank, and Mr. E. O. Phillipi; £100 each from Mr. Daniel Coats, Mr. and Mrs. John Thomson, Dowanhill; Messrs. Greenlees and Sons, Possil Park; Mrs. Cargill, Glasgow; Messrs. Denny and Co., Dumbarton; Mr. George A. Mitchell, Glasgow; and Dr. and Mrs. Parker, Kilmacolm.

### THE SATURDAY FUND'S SUCCESS.

It is most gratifying to learn of the continued prosperity of the Hospital Saturday Fund, which was revealed in a remarkable degree at the annual meeting held at the Mansion House last week. The income for 1915 is almost £7,000 above that received in 1914, and even surpasses the 1913 total. In spite of innumerable members having been called to the Colours, in spite of the operations of the Insurance Act, the prosperity of the Hospital Saturday Fund is maintained and increased. That this is no exaggeration, or momentary phase, is apparent from the fact that during the first three months of the present year £2,530 has been collected over and above the total raised during the same period twelve months ago. War work, wages, and the full realisation of what hospital work can do help to account for this. It is, therefore, most urgently to be hoped that the Government are *not* adopting a "wait-and-see" policy in regard to the transition to peace; for, if any grave dislocation of industrial conditions be allowed to arise after hostilities are ended, the great funds, of which the Hospital Saturday Fund is the type and example, will run a grave risk of being engulfed in the crisis that, unless prepared for and guarded against, is virtually certain to occur.

### THE SOUTHERN HOSPITAL, LIVERPOOL, AND A LOCAL WAGER.

A sporting episode has just taken place which promises in the end to benefit the funds of the Southern Hospital, Liverpool, to a small but not negligible degree. At a meeting of the British Empire Union lately held in that city, Mr. Kebty-Fletcher criticised very strongly certain views expressed by Sir William Lever at the annual meeting of the firm which he founded. He offered to bet Sir William fifty pounds to a penny that eighteen months after the war the German mark would fall another 20 per cent. beyond its present depreciated level, and that labour in Germany would not command more than twenty marks a week. Sir William Lever has accepted both bets. He wrote to the editors of the *Courier* and the *Daily Post and Mercury*, enclosing with each letter a penny stamp as his stake, with a request in each case that the penny should be handed to Mr. Kebty-Fletcher if he wins, and that in the opposite event the latter's stake should go to the hospital. Not to be outdone, Mr. Kebty-Fletcher announces that if he wins, not only shall the hospital have his winnings (of twopence), but that he will also give 2,500 pennies as well. The Southern Hospital thus stands to gain either a hundred pounds or ten pounds eight shillings and sixpence from this sporting wager.

### MEDICINAL HERB CULTIVATION IN HOSPITAL GARDENS.

The extreme scarcity of some of our most valuable medicinal herbs, which has resulted from the cessation of supplies from Central Europe, has stimulated the desire among a section of the community to make this country as far as possible independent of foreign sources. Unfortunately, however, there seem to exist a good many false notions concerning the industry of herb farming and its prospects, and such lectures as the one delivered by Mr. E. M. Holmes at a meeting of the Royal Horticultural Society on April 11 should go some way towards removing erroneous impressions and directing efforts into the right channel. Mr. Holmes is recognised as one of the great authorities on the cultivation of herbs, and in his own experimental garden has grown nearly every herb that can be cultivated in this climate. His view is that while, generally speaking, the cultivation of medicinal plants can only be carried on profitably in cases where the herb farm is worked in connection with a pharmaceutical factory, we could render ourselves independent of Germany and Austria for our supply of many herbs if a system of collecting, drying, and distributing them were properly organised. As for belladonna and digitalis, he thinks that if the head-gardeners on large estates were to pay a little intelligent attention to these plants, this country would soon be able not only to satisfy its own requirements, but would have a surplus of supplies for export. Mr. Holmes, in his lecture, did not suggest the utilisation of hospital gardens for the growing of medicinal herbs, but it would certainly seem appropriate to use land adjoining hospitals for this purpose.

### SCHOOL INSPECTION IN LEEDS.

Considering the difficulties which were imported into the work of school inspection last year, the report on the condition of the school children of Leeds is evidence of very creditable work. Dr.

Lee Bolton has acted as S.M.O. to the Leeds Education Committee in the absence of Dr. A. Wear on active service, two assistant medical officers being similarly engaged elsewhere. With the help of local practitioners nearly the whole of the service has been carried on, and over 17,000 children examined. Among the points of interest in this exhaustive report may be mentioned the fact that scholars coming from the poorer districts showed a lower percentage with dental caries than those attending schools in better districts; while, curiously enough, in the Jewish schools the percentage is higher still. The figures indicate, however, a highly satisfactory reduction all round, which tends to show that greater interest in the care of the teeth is being taken by the scholars. The principal reasons for the rejection of recruits at the present time are those relating to defects such as are being discovered and rectified by the medical inspectors of schools, and hence the continuance of this work is of paramount importance to the nation. Some idea of the scope of the work in a large town like Leeds may be gathered from the fact that during the year 800,000 attendances had been lost owing to necessary exclusions, and this involved a decrease in grants of about £4,166. In the future attendances at the school clinics will be allowed to count as school attendances, and thus the financial loss will be mitigated.

### X-RAY ASSISTANTS.

The question whether a nurse can properly undertake the duties of assistant in an *x*-ray department was raised recently before the Lambeth Tribunal, when the authorities of King's College Hospital appealed for the exemption of Mr. A. O. Forder, an assistant in the *x*-ray department. It was urged that the man was indispensable, but the Tribunal pointed out that at some institutions the nurses did this work. On behalf of King's College, it was admitted that nurses could do the work, but it was also urged that it would take a long while to train them. This man had to take care of the apparatus, which was very complicated and which was a dangerous thing if not properly handled. At the present time it was quite impossible to obtain a substitute if the man were ordered to join the Army. The Tribunal granted three months' exemption.

### SOME MASSAGE HOWLERS.

At the January and February examination of the Incorporated Society of Trained Masseuses, 159 candidates sat for the Society's certificate and 124 passed. The markers of the papers report that a few candidates revealed somewhat extraordinary methods of expression. One student advocated "plenty of arm and leg shaking to increase the supply of nourishment to the tissues and shake the red marrow up in the bones," and another, in explaining what she would do in certain circumstances, said, "Then I should give breathing, as this contracts the diaphragm and causes it to act as an internal masseuse," but on the whole the papers showed care and thoughtfulness. At a practical examination for fifteen male candidates fourteen of the men were blind, and the only failure was the sighted student. Candidates at the examinations were received from seventeen hospitals and infirmaries, fifteen private schools, and eight private teachers.

### WHERE TO TREAT OUR COLONIAL WOUNDED.

Sympathy and patriotism were aroused so spontaneously on the offers of help from the Colonies arriving here, that the arrival of the first wounded from the Dominion Forces was an event of historic importance. We could not do too much for him. It was quickly suggested that he would be happier surrounded by patients, doctors, and nurses from his own country, and New Zealand, Australian, and Canadian hospitals sprang up everywhere. Now that these are in full swing it is suggested that the plan of segregation has its disadvantages. The Colonial, we are told, likes to be in one of the ordinary hospitals of the Old Country; he likes the opportunity of fraternising with us; of making new friends with us, of getting to know the relatives of his cousins over here. It is also pointed out, no doubt justly, that the advantages our "Tommies" get from meeting the Colonial are very great. As he has taught the Old Country what the Empire means to him, so it is said he teaches many a Cockney what regard for the Old Country can move the hearts of those who have left her for daughter lands. The effect of such friendships and "mixing" on the stream of emigrants which will begin with Peace, is likely to be immense. Why, then, not check the tendency to segregation by limiting the growth of the "Dominion" hospitals? It is so easy to argue for, or against, the proposal that we do not intend to do so. The advantages of "mixing" are so many that, provided the wish of any Colonial to be in familiar surroundings is not discounted, we hope that the administrative advantages of segregation will not be allowed to stand in the way.

### THIS WEEK'S DRUG MARKET.

Business in the drug market has become much quieter, and until the Easter holidays are over it will remain quiet. Price changes have been less numerous than usual, but on the whole values are fairly well maintained. Fabulous prices are now quoted for cod-liver oil, with the result that no business is being done for home consumption; in view of the extraordinary figures Norwegian producers are asking it is clearly necessary for prescribers to order cod-liver oil as rarely as possible. Tartaric acid and citric acid still have an upward tendency in price, and it seems likely that the values may be still higher owing to the difficulties of obtaining supplies. Cream of tartar is slightly dearer. Sugar of milk has an upward tendency in price. In synthetic drugs there are few price changes of any note, but the value of phenacetin still tends upwards, while the prices of phenazone and acetanilide are not quite so firmly maintained. There is no quotable change in bromides, but, if anything, the tendency is in a downward direction.

